# Activation of *Drosophila melanogaster* TRPA1 Isoforms by Citronellal and Menthol

**DOI:** 10.3390/ijms222010997

**Published:** 2021-10-12

**Authors:** Brett Boonen, Justyna B. Startek, Alina Milici, Alejandro López-Requena, Melissa Beelen, Patrick Callaerts, Karel Talavera

**Affiliations:** 1 Leuven Center for Brain & Disease Research, Laboratory of Ion Channel Research, Department of Cellular and Molecular Medicine, KU Leuven, VIB-KU 3000 Leuven, Belgium; brett.boonen@kuleuven.be (B.B.); justyna.startek@kuleuven.be (J.B.S.); alina.milici@kuleuven.be (A.M.); alopezr1@yahoo.com (A.L.-R.); 2 Laboratory of Behavioral and Developmental Genetics, Department of Human Genetics, KU Leuven, 3000 Leuven, Belgium; melissa.beelen@kuleuven.be (M.B.); patrick.callaerts@kuleuven.be (P.C.)

**Keywords:** TRPA1, *Drosophila melanogaster*, isoform, citronellal, menthol, AITC, HC-030031, repellent, avoidance, olfaction

## Abstract

Background: The transient receptor potential ankyrin 1 (TRPA1) cation channels function as broadly-tuned sensors of noxious chemicals in many species. Recent studies identified four functional TRPA1 isoforms in *Drosophila melanogaster* (dTRPA1(A) to (D)), but their responses to non-electrophilic chemicals are yet to be fully characterized. Methods: We determined the behavioral responses of adult flies to the mammalian TRPA1 non-electrophilic activators citronellal and menthol, and characterized the effects of these compounds on all four dTRPA1 channel isoforms using intracellular Ca^2+^ imaging and whole-cell patch-clamp recordings. Results: Wild type flies avoided citronellal and menthol in an olfactory test and this behavior was reduced in *dTrpA1* mutant flies. Both compounds activate all dTRPA1 isoforms in the heterologous expression system HEK293T, with the following sensitivity series: dTRPA1(C) = dTRPA1(D) > dTRPA1(A) ≫ dTRPA1(B) for citronellal and dTRPA1(A) > dTRPA1(D) > dTRPA1(C) > dTRPA1(B) for menthol. Conclusions: *dTrpA1* was required for the normal avoidance of *Drosophila melanogaster* towards citronellal and menthol. All dTRPA1 isoforms are activated by both compounds, but the dTRPA1(B) is consistently the least sensitive. We discuss how these findings may guide further studies on the physiological roles and the structural bases of chemical sensitivity of TRPA1 channels.

## 1. Introduction

TRPA1 is a Ca^2+^-permeable non-selective cation channel found in vertebrates and invertebrates [1,2,3]. All TRPA1 orthologues share the characteristic of functioning as polymodal sensors, being activated by physical and chemical stimuli [4,5]. In close analogy to TRPA1 localization in vertebrate sensory neurons acting as noxious chemosensors [6,7,8,9], dTRPA1 is localized in bitter-sensing gustatory neurons [4,10] and olfactory neurons [11]. Activation of TRPA1 in these tissues is perceived as a noxious sensation that results in nocifensive behaviors, such as immediate avoidance [12,13,14,15,16]. Some chemosensory properties of TRPA1 channels are conserved across several hundreds of millions of years of evolution. This is illustrated by the sensitivity of insect and mammalian TRPA1 orthologous to noxious electrophilic compounds (e.g., allyl isothiocyanate, AITC) [4,6,7,9] and to bacterial lipopolysaccharides [16,17,18,19,20]. This conservation enables the use of the fruit fly *Drosophila melanogaster* as a relevant model for studying several aspects of TRPA1 pharmacology, physiology and pathophysiology. However, some striking differences between insect and mammalian TRPA1 orthologues have been reported. First, mammals express only one functional TRPA1 isoform, while to date up to four functional TRPA1 isoforms have been identified in *Drosophila* (dTRPA1(A), (B), (C) and (D)) [21,22]. Mouse TRPA1 was shown to be activated by cold [8,23,24], the human orthologue is activated by both heating and cooling [25], whereas dTRPA1 was shown to be heat-activated and to be implicated in noxious heat sensing [12,15,26]. Moreover, the physiological roles and the sensitivity to heat have been shown to vary across different dTRPA1 isoforms [10,21].

The sensitivity of TRPA1 channels to electrophilic chemicals is largely conserved along evolution [4], but in contrast, notably different sensitivities to non-electrophiles among TRPA1 orthologues have been reported. One of the most prominent examples is menthol, the component responsible for the fresh sensation of mint [27,28,29,30,31], which was shown to activate both mouse and human TRPA1 [31,32], but to be ineffective on dTRPA1 [4,33]. These initial observations complicate the interpretation of a subsequent study reporting that dTRPA1 is implicated in nocifensive rolling responses of *Drosophila* larvae to topical application of menthol [34]. Another example of non-electrophile reported to have species-dependent effects is citronellal, well-known insect repellent [35]. This compound was shown to activate human TRPA1 [14], but to weakly and indirectly activate dTRPA1 via a G-protein/PLC-coupled transduction pathway [11]. Later, citronellal was shown to facilitate gustatory feeding aversion via a direct activation of dTRPA1(A) [14], and also to activate dTRPA1(C) and dTRPA1(D) heterologously expressed in *Xenopus laevis* oocytes [36]. Some of these differences across species have been exploited to identify critical regions for pharmacological TRPA1 modulation [33,37], which is crucial for the rational design of therapeutic strategies targeting this channel.

Many of these studies were performed prior to the discovery of the different dTRPA1 isoforms and thus focused on the properties of only one or two isoforms. Furthermore, the use of multiple experimental settings complicates any systematic comparison of the pharmacological properties of the different dTRPA1 isoforms. In order to address these issues, in this study we characterized the chemical sensitivities of the four dTRPA1 isoforms (dTRPA1(A), (B), (C) and (D)). We focused on the non-electrophilic compounds citronellal and menthol because the four dTRPA1 isoforms have been shown to respond similarly to the electrophilic agonist AITC [21,38].

We first studied the aversive responses of adult *Drosophila melanogaster* flies to citronellal and menthol. Elucidating the mechanisms of the behavioral effects of these compounds, including the implications of dTRPA1 and alternative pathways, helps understanding their relevance in nature (e.g., plant herbivore interactions [39]), and may serve to optimize their use as pest control [11]. We took advantage of the volatile nature of citronellal and menthol to assess the fly avoidance behavior using an adapted direct airborne repellent test. We found that dTRPA1 channels are necessary for the avoidance response of flies to these compounds. Subsequently, we tested whether these in vivo effects could be mediated by activation of dTRPA1 by characterizing the responses of the different channel isoforms using HEK293T cells as heterologous expression system. These experiments yielded that all four dTRPA1 isoforms can be activated by citronellal and menthol, albeit with markedly different sensitivities.

## 2. Results

### 2.1. dTrpA1 Is Implicated in the Avoidance of Citronellal and Menthol by Drosophila melanogaster

**Figure 1:**
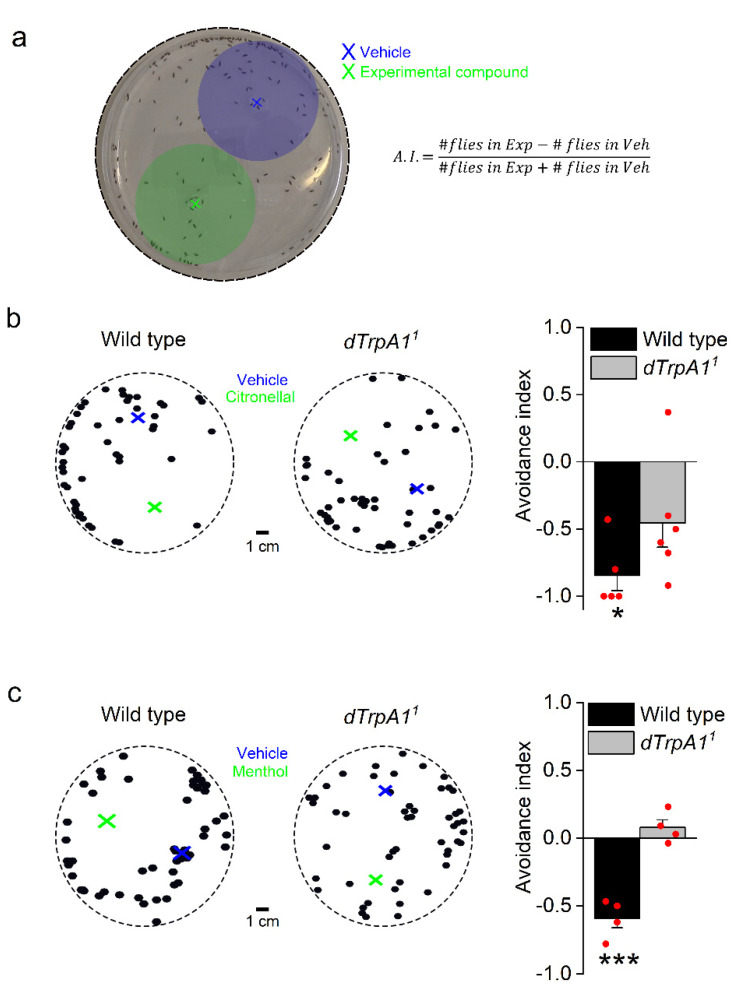
Adult *Drosophila melanogaster* flies avoid citronellal and menthol in a *dTrpA1*-dependent manner. (**a**) Direct airborne repellent test. Thirty minutes after loading the vehicle and the test solutions at the locations indicated by the blue and green crosses respectively, the flies found in vehicle and test areas (marked by the blue and green shades) were counted and the avoidance index (A.I.) was calculated using the formula shown on the right. #Flies in Exp and #Flies in Veh stand for the number of flies found in the regions corresponding to the experimental compound and vehicle, respectively. Positive values of A.I. indicate preference and negative values indicate avoidance. (**b**) Examples of the positions of WT (**left**) and *dTrpA1^1^* (**right**) flies exposed to vehicle (blue; 20 µL DMSO) and citronellal solutions (green; 20 µL of 1 mM citronellal in DMSO). The bar graph on the right shows the average A.I. to citronellal for WT flies (*n* = 5 plates, one-sample *t*-test vs. 0, *p* = 0.026 indicated with *) and *dTrpA1^1^* flies (*n* = 6 plates, one-sample *t*-test vs. 0, *p* = 0.4). WT vs. *dTrpA1^1^*, two sample *t*-test, *p* = 0.26. (**c**) Examples of WT (**left**) and *dTrpA1^1^* (**right**) fly positions after exposure to vehicle (blue; 20 µL ethanol) and the menthol solutions (green; 20 µL of 1 mM menthol in ethanol). The bar graph on the right displays the average A.I. to menthol for WT (*n* = 4 plates, one-sample *t*-test vs. 0, *p* = 0.004 indicated by ***) and *dTrpA1^1^* flies (*n* = 4 plates, one-sample *t*-test vs. 0, *p* = 0.26). WT vs. *dTrpA1^1^*, two sample *t*-test, *p* = 0.0004.

As both citronellal and menthol are volatile, we assessed *D. melanogaster* behavior in an olfactory-based assay and compared wild type (WT) and *dTrpA1*-deficient (*dTrpA1^1^*) flies. We used an adapted version of a previously described direct airborne repellent test (DART) [11] and analyzed the behavior of the flies in terms of the avoidance index (A.I., see Materials and methods and Figure 1a). We found that WT flies avoided citronellal and that this response was significantly lower in *dTrpA1*-deficient flies (Figure 1b, Supplemental Figures S1 and S2 and Supplemental Videos S1 and S2). These results agree with previous observations [11] and thus validate the assay for evaluating odor-based avoidance.

Adult female *Drosophila* flies avoid menthol-spiked surfaces during oviposition [40] and larvae display nocifensive behavior towards this compound in a *dTrpA1*-dependent manner [34]. Using our variant of the DART assay, we found that adult WT *Drosophila* flies avoid menthol (Figure 1c and Supplemental Figures S1 and S2 and Supplemental Videos S3 and S4). In contrast, this behavior is completely absent in *dTrpA1^1^* flies (Figure 1c). Thus, lack of dTRPA1 leads to impaired avoidance of both citronellal and menthol, but the impact of *dTrpA1* deletion is strikingly stronger for the latter compound. The implication of *dTrpA1* in the responses to citronellal and menthol led us to re-examine whether these compounds activate dTRPA1 channels. To do so, we used whole-cell patch-clamp electrophysiology and intracellular Ca^2+^ imaging in the HEK293T heterologous expression system.

### 2.2. Basal Currents in HEK293T Cells Transfected with dTRPA1 Isoforms

**Figure 2:**
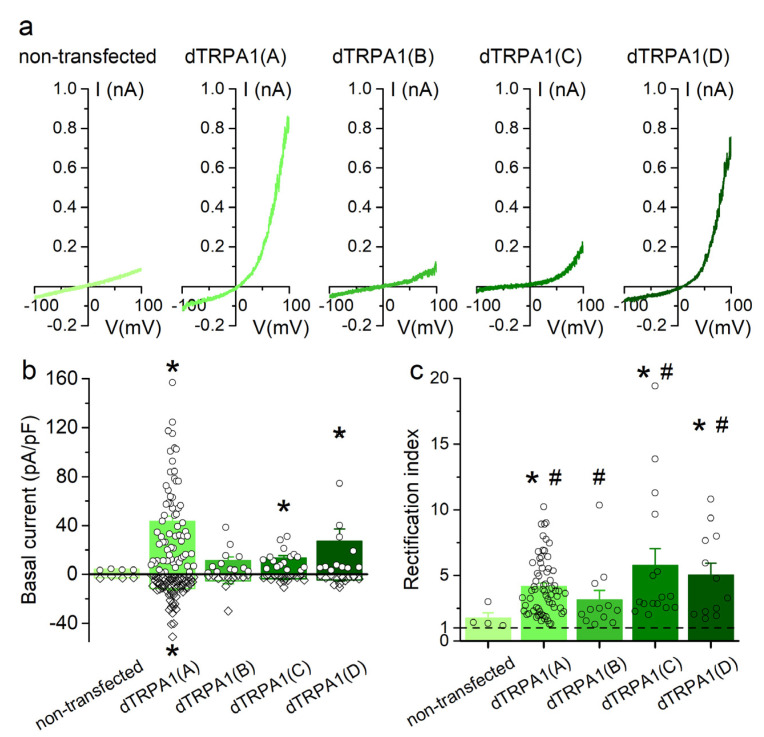
Basal currents recorded in HEK293T cells expressing dTRPA1 isoforms. (**a**) Examples of basal (non-stimulated) currents recorded in non-transfected cells and in cells expressing either of the four dTRPA1 channel isoforms. (**b**) Average basal current density determined at +75 mV (filled bars) and −75 mV (open bars) in non-transfected and in cells expressing dTRPA1(A), dTRPA1(B), dTRPA1(C) or dTRPA1(D) (n = 4, 66, 12, 16 and 12, respectively). (**c**) Average rectification index determined for the same cells as in (**b**). The * symbols indicate *p* < 0.05 compared to non-transfected cells, with Kruskal-Wallis test. The # symbols indicate *p* < 0.05 compared to 1, with Wilcoxon signed rank test.

We first tested whether the dTRPA1 isoforms display constitutive activity in HEK293T cells by comparing the basal (non-stimulated) currents recorded in non-transfected cells to those recorded in cells transfected with each of the four dTRPA1 isoforms. Currents recorded in non-transfected cells were of very small amplitude (Figure 2a,b) and displayed a rather linear voltage dependence (rectification index calculated as –I(+75 mV)/I(−75 mV) not significantly different from 1; Figure 2a,c). This is consistent with a lack of endogenous functional expression of TRPA1 channels in HEK293T cells, as previously shown [41,42].

dTRPA1(A)-transfected cells displayed robust outward and inward currents, whose amplitudes at −75 and +75 mV were significantly larger than those recorded in non-transfected cells (Figure 2a,b). These currents were outwardly rectifying (rectification index ~4; Figure 2c).

dTRPA1(B)-transfected cells showed currents that were not significantly different in amplitude and rectification index with respect to non-transfected cells (Figure 2a–c). However, the rectification index was statistically significantly higher than 1 (Figure 2c), indicating outward rectification. The currents recorded at −75 mV in cells transfected with dTRPA1(C) or dTRPA1(D) were not significantly larger than in non-transfected cells, but outward currents were significantly larger (Figure 2a,b), which resulted in larger rectification indexes (~6 and ~5, respectively; Figure 2c). These data show that isoforms A, C and D display constitutive activity when heterologously expressed in HEK293T cells. For the B isoform, the evidence of constitutive activity is limited to the finding that the currents showed significant outward rectification, in contrast to the currents recorded in non-transfected cells.

### 2.3. Effects of Citronellal on dTRPA1 Channel Isoforms

The effects of citronellal were previously tested in all dTRPA1 isoforms, but this was done in two separate studies that used *Xenopus laevis* oocytes as expression system [14,36]. We tested all four isoforms for direct comparison using intracellular Ca^2+^ imaging experiments in HEK293T cells. We determined the responses of non-transfected and dTRPA1-transfected cells to citronellal applied cumulatively in increasing concentrations (0.001–1 mM) because, as argued before [43], this stimulation resembles closely the in vivo situation, in which concentration increases gradually at the sensory cells.

**Figure 3:**
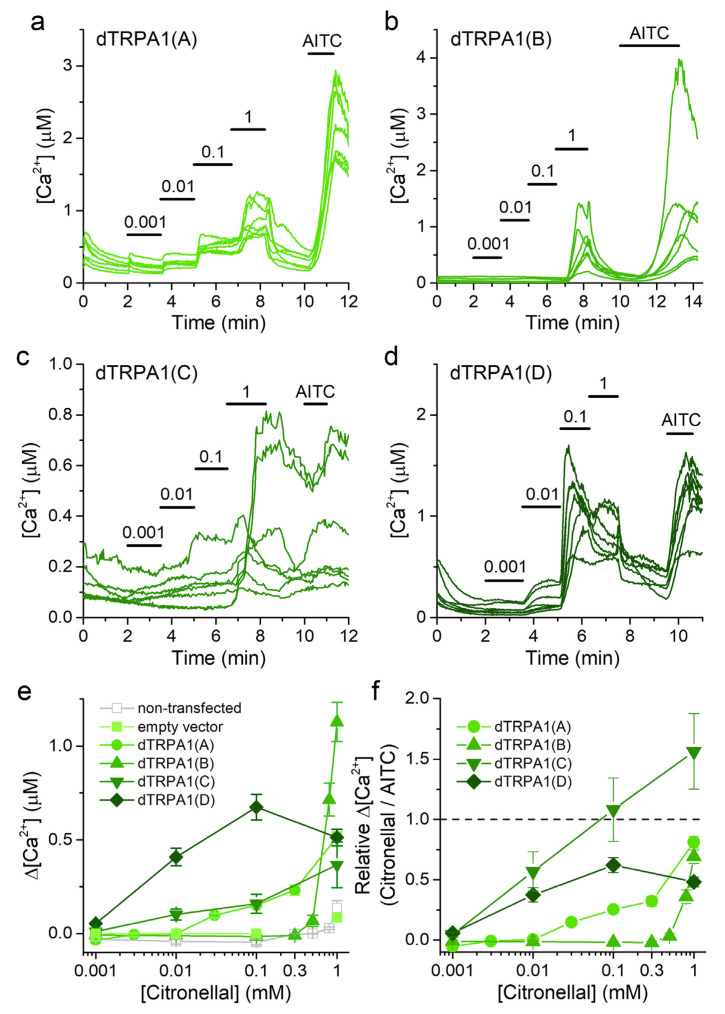
Citronellal increases intracellular [Ca^2+^] in cells transfected with dTRPA1 isoforms. (**a**–**d**) Example traces of changes of intracellular Ca^2+^ concentration induced by citronellal (0.001–1 mM) and AITC (100 µM) in HEK293T cells expressing either dTRPA1(A), dTRPA1(B), dTRPA1(C) or dTRPA1(D). (**e**,**f**) Concentration dependence of the average amplitude of intracellular Ca^2+^ increase induced by citronellal; panel (**e**) raw data and panel (**f**) data obtained by normalizing to the amplitude of the response to AITC in each cell. *n* = 102–298, 21–75, 30 and 39 for isoforms A to D, respectively.

All isoforms responded to citronellal (Figure 3a–d) in a concentration-dependent manner (Figure 3e). Non-transfected HEK293T cells and cells transfected with the empty vector responded only weakly to 1 mM citronellal (Figure 3e), indicating that the responses in transfected cells were largely mediated by the respective dTRPA1 channels. However, these endogenous Ca^2+^ responses of HEK293T cells prevented us from confidently testing the effects of citronellal at concentrations higher than 1 mM on the dTRPA1 channels. As a consequence, it was impossible to fit the data to estimate the effective stimulatory concentrations (EC_50_) because none of the concentration dependencies obtained in the transfected cells showed a clear saturation behavior at the highest concentration tested. To circumvent this limitation, we used the minimal concentration at which the Ca^2+^ increase was detectable as a surrogate measure for the sensitivity to citronellal (tested as significant difference above the baseline Ca^2+^ level with a One Sample Wilcoxon Signed Rank test). They were thus, in decreasing order: dTRPA1(C) = dTRPA1(D) > dTRPA1(A) ≫ dTRPA1(B) (Figure 3e).

Attending to their average maximal amplitudes, the responses of transfected cells to citronellal decreased in the following order: dTRPA1(B) > dTRPA1(A) = dTRPA1(C) = dTRPA1(D) (Figure 3e). As a way to compare the effects of citronellal to those of the strong TRPA1 agonist AITC we re-plotted the concentration-dependence curves but using the amplitudes of the responses to citronellal normalized to the amplitude of the subsequent response to AITC (100 µM) in each cell (Figure 3f). These normalized data show that citronellal induces weaker responses than AITC for all dTRPA1 isoforms except for dTRPA1(C), for which the responses to 0.1 and 1 mM citronellal were similar or larger than for AITC, respectively. Interestingly, in cells transfected with dTRPA1(D) the Ca^2+^ increase induced by 0.1 mM citronellal exceeded the response elicited by 1 mM citronellal (Figure 3e). This might be caused by partial desensitization during the cumulative application of the compound (Figure 3d).

**Figure 4:**
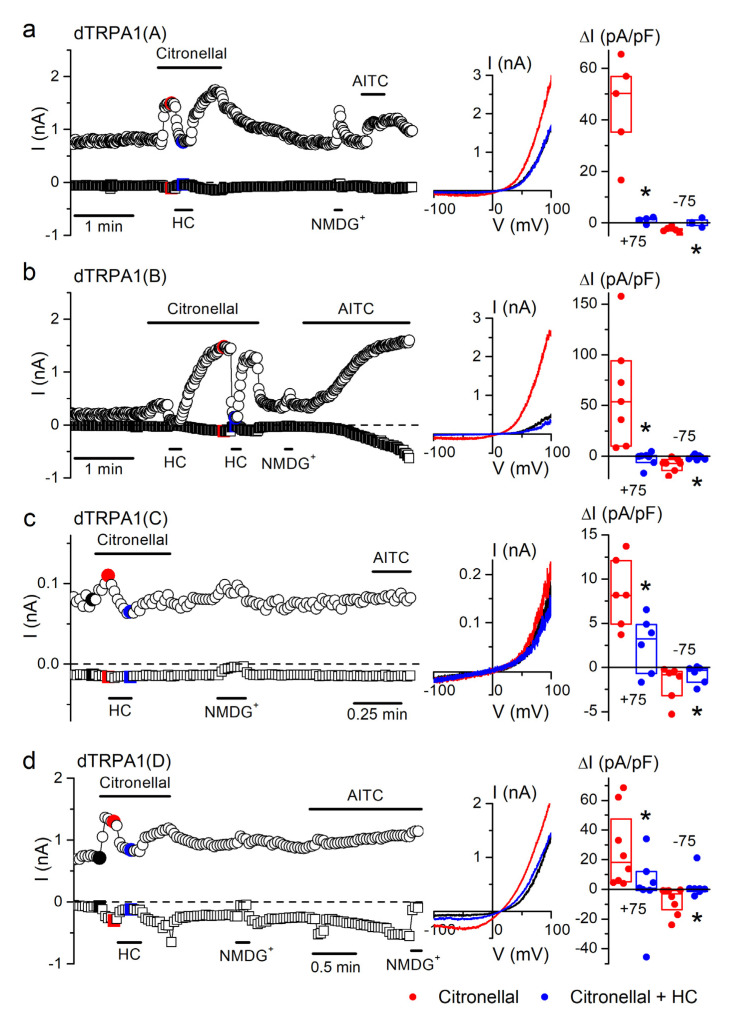
Citronellal increases whole-cell currents in the four dTRPA1 isoforms. (**a**–**d**) Examples of the effects of citronellal (1 mM), HC-030031 (HC; 100 µM) and AITC (100 µM) on the currents recorded at +75 mV (circles) and −75 mV (squares) in whole-cell patch-clamp experiments in HEK293T cells expressing either dTRPA1(A), dTRPA1(B), dTRPA1(C) or dTRPA1(D). A solution where all cations were replaced by N-methyl-D-glucamine (NMDG^+^) was used to assess the presence of leak currents [44]. The middle insets represent current traces recorded at the time points indicated by the corresponding colors in the left panels. The dot plots on the right represent the change in current density amplitude with respect to baseline values (recorded at +75 and −75 mV). The horizontal lines represent medians and the boxes represent the 25–75 percentiles. Application of citronellal (1 mM, red symbols) induced whole-cell current increases in cells expressing: dTRPA1(A) (*p* = 0.003, *n* = 5), dTRPA1(B) (*p* = 0.01, *n* = 7), dTRPA1(C) (*p* = 0.0016, *n* = 6) or dTRPA1(D) (*p* = 0.01, *n* = 8, one-sample *t*-test vs. 0). The TRPA1 blocker HC-030031 (HC; 100 µM) inhibited citronellal-induced (1 mM) currents (blue symbols) in cells expressing dTRPA1(A) (*p* = 0.003, *n* = 4–5), dTRPA1(D) (*p* = 0.024, *n* = 7–8; two-sample *t*-test), dTRPA1(C) (*p* = 0.00006, *n* = 6) or dTRPA1(B) (*p* = 0.018, *n* = 7; one tailed paired sample *t*-test).

Whole-cell patch-clamp experiments confirmed that all dTRPA1 isoforms are rapidly and reversibly stimulated by 1 mM citronellal (Figure 4a–d). The maximal outward currents evoked at +75 mV by this compound were highest for dTRPA1(B), followed by dTRPA1(A), dTRPA1(D) and much smaller for dTRPA1(C). Maximal inward currents stimulated by citronellal at −75 mV were largest for dTRPA1(B) and dTRPA1(D), followed by dTRPA1(A) and dTRPA1(C). It must be noted that cells transfected with dTRPA1(C), as assessed from the green fluorescence emission conferred by the expression of GFP, were often insensitive to both citronellal and AITC (data not shown). Application of 100 µM HC-030031 during citronellal exposure reduced both inward and outward currents to near basal levels for dTRPA1(A), dTRPA1(B) and dTRPA1(D), and had weaker effects on the amplitudes of currents recorded in cells transfected with dTRPA1(C) (Figure 4a–d).

### 2.4. Effects of Menthol on dTRPA1 Channel Isoforms

Menthol was previously reported to have no effect on the dTRPA1(B) isoform heterologously expressed in HEK293T cells [33]. We used the same expression system to characterize the responses of the four dTRPA1 isoforms to this compound (0.3–1 mM) using Ca^2+^ imaging. 

**Figure 5:**
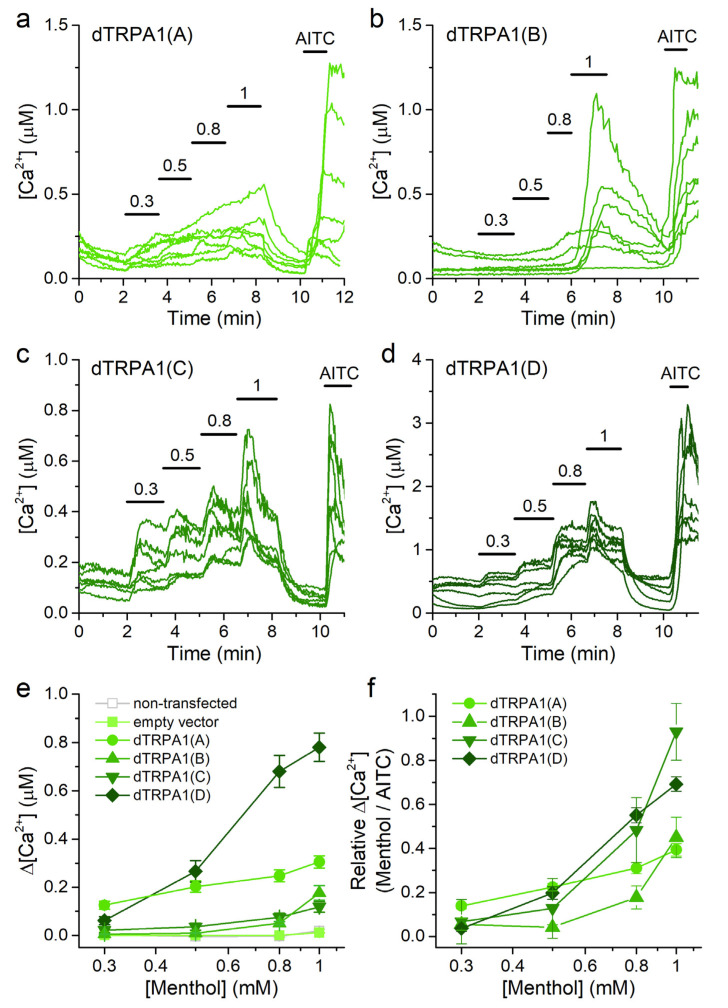
Menthol increases intracellular Ca^2+^ in cells transfected with dTRPA1 isoforms. (**a**–**d**) Example traces of changes of intracellular Ca^2+^ concentration induced by menthol (0.3–1 mM) and AITC (100 µM) in HEK293T cells expressing: dTRPA1(A), dTRPA1(B), dTRPA1(C) or dTRPA1(D). (**e**,**f**) Concentration dependence of the average amplitude of intracellular Ca^2+^ increase induced by menthol; panel (**e**) raw data and panel (**f**) data obtained by normalizing to the amplitude of the response to AITC in each cell. *n* = 127, 210, 43 and 65 for isoforms A to D, respectively.

We found that HEK293T cells transfected with either of all dTRPA1 isoforms displayed rises in intracellular Ca^2+^ concentration upon stimulation with menthol at increasing concentrations (Figure 5a–d). In contrast, neither non-transfected HEK293T cells nor cells transfected with the empty vector respond to menthol when applied at concentrations up to 1 mM (Figure 5e). We did not apply menthol above this concentration because of previous reports demonstrating endogenous Ca^2+^ release from intracellular stores [45]. This limited the possibility of reaching maximal dTRPA1 activation and precluded the accurate determination of the EC_50_ values. As for the analysis of data on citronellal, we therefore estimated the sensitivity to menthol from the minimal concentration at which the Ca^2+^ increase was detectable (tested as significant difference above the baseline Ca^2+^ level with a one sample Wilcoxon signed rank test). As such, the sensitivity series was dTRPA1(A) > dTRPA1(D) > dTRPA1(C) > dTRPA1(B) (Figure 5e). The maximal amplitude of the responses to menthol followed the series: dTRPA1(D) > dTRPA1(A) > dTRPA1(B) = dTRPA1(C) (Figure 5e). The concentration dependencies normalized to the amplitude of the responses to AITC revealed that the responses of dTRPA1(C) to 1 mM menthol were comparable to the responses to AITC, whereas the responses of dTRPA1(A), dTRPA1(B) and dTRPA1(D) were smaller (Figure 5f).

Menthol has a bimodal effect on mouse TRPA1, inducing activation at low concentrations, inhibition at high concentrations, and a subsequent rebound of activity upon washout [31]. In contrast, we found that none of the dTRPA1 isoforms displayed inhibition at high menthol concentrations. Furthermore, we did not observe rebound effects, as upon washout the Ca^2+^ levels of all dTRPA1-expressing cells reversed monotonically to near baseline levels within a couple of minutes, regardless of the isoform expressed (Figure 5a–d).

**Figure 6:**
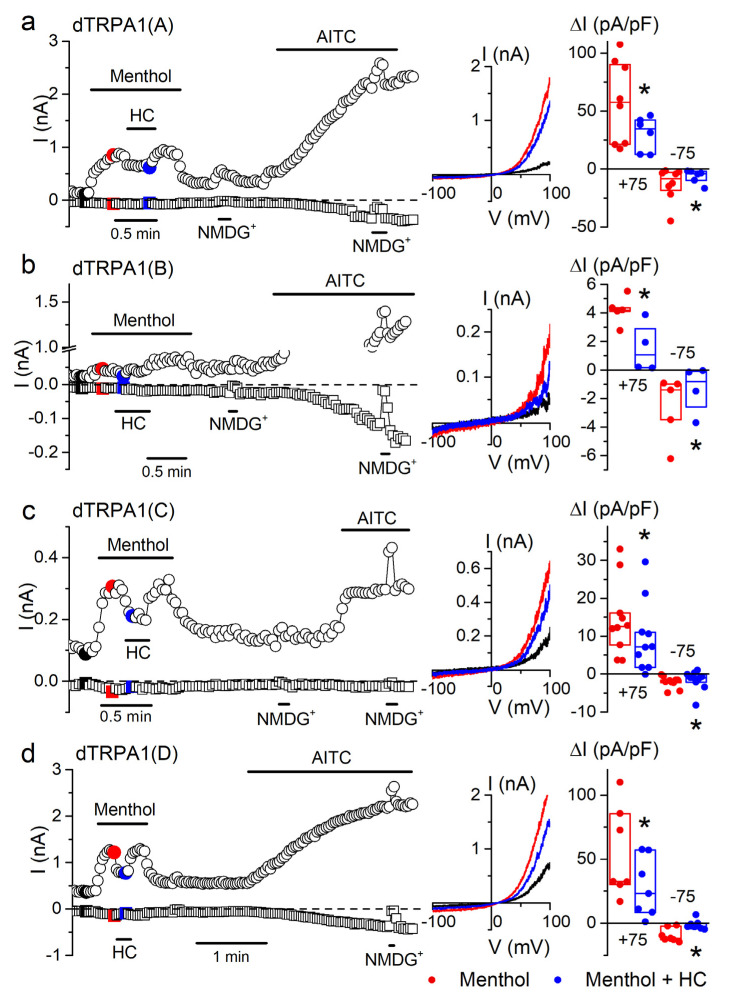
Menthol increases whole-cell currents in the four dTRPA1 isoforms. (**a**–**d**) Example of the effects of menthol (1 mM), HC-030031 (HC; 100 µM) and AITC (100 µM) on the amplitude of whole-cell currents measured at +75 mV (circles) and −75 mV (squares) in HEK293T cells expressing either dTRPA1(A), dTRPA1(B), dTRPA1(C) or dTRPA1(D). An extracellular solution where all cations were replaced by *N*-methyl-d-glucamine (NMDG^+^) was used to assess the size of leak currents. The middle insets show current traces recorded at the time points indicated by the corresponding colors in the left panels. The dot plots on the right show the change in current density amplitude with respect to baseline values (+75 mV and −75 mV). The horizontal lines represent the medians and the boxes represent the 25–75 percentiles. Application of menthol (1 mM, red symbols) increased whole-cell current in cells expressing: dTRPA1(A) (*p* = 0.001, *n* = 8), dTRPA1(B) (*p* = 0.0003, *n* = 5), dTRPA1(C) (*p* = 0.0006, *n* = 10) or dTRPA1(D) (*p* = 0.003, *n* = 7, one-sample *t*-test vs. 0). The TRPA1 blocker HC-030031 (HC; 100 µM) partly inhibited menthol-induced currents (blue symbols) in cells expressing dTRPA1(A) (*p* = 0.04, *n* = 6–8), dTRPA1(B) (*p* = 0.024, *n* = 4–5; two-sample *t*-test), dTRPA1(C) (*p* = 0.0004, *n* = 10) or dTRPA1(D) (*p* = 0.004, *n* = 7; one tailed paired sample *t*-test). * *p* < 0.05.

We confirmed the activation of all dTRPA1 isoforms by menthol (1 mM) using whole-cell patch-clamp recordings (Figure 6). The maximal amplitude of outward currents evoked by menthol were the highest for dTRPA1(A) and dTRPA1(D), followed by dTRPA1(C) and were the lowest for dTRPA1(B). Menthol-induced currents were reversibly blocked by the application of the TRPA1 inhibitor HC-030031 (100 µM; Figure 6a–d). However, the inhibitory effects of this compound on the menthol-induced currents were weaker than on those elicited by citronellal. Taken together, our results show that all dTRPA1 isoforms respond to menthol in a concentration-dependent and reversible manner.

## 3. Discussion

Since the first descriptions of TRPA1 as a target of noxious chemicals [6,9] the list of compounds reported to activate this channel has grown rapidly [1,2,3,46]. The distinct pharmacological properties of TRPA1 homologues from different species has served in structure-function studies aimed at elucidating the molecular determinants underlying this impressive chemical promiscuity [33,37]. Because multiple studies on *Drosophila* TRPA1 were performed prior to the identification of the different isoforms of this channel [21], it remained unclear to what extent these isoforms have similar chemical sensitivities. The available data suggests that all four isoforms are similarly activated by AITC [21,38]. The effects of non-electrophilic compounds on all four isoforms remained yet to be determined.

In this study, we focused on the non-electrophiles citronellal and menthol, as they have been shown to trigger avoidance responses in *Drosophila melanogaster* [11,14,34,40]. Previous results on the implication of dTRPA1 in olfactory-mediated behaviors were obtained with a DART that utilized a quasi-linear arena in which flies were contained in a long tube. It was shown that WT flies avoided citronellal and that this response was significantly lower but not completely abrogated in *dTrpA1*-deficient flies [11]. We here used a variant of the DART using a circular arena, which arguably results in less crowding effects. We obtained results similar to those previously reported [11], indicating that our settings were appropriate to study olfactory-mediated behaviors. Moreover, we reinforce the notion that other receptors distinct from dTRPA1 may mediate part of the aversive responses of *D. melanogaster* to citronellal. These include the olfactory coreceptor OR83b [11], and potentially, 5-HT and GABA receptors [47,48,49].

We then characterized the responses of the A, B, C and D dTRPA1 channel isoforms to these compounds. Because no native experimental model is currently available for direct functional characterization, we assessed the properties of dTRPA1 isoforms using HEK293T cells as an expression system. On one hand, this might limit the reach of our results to the understanding of the relevance of dTRPA1 channels in vivo, which is nevertheless inherent to all heterologous and artificial expression systems. On the other hand, HEK293T cells have been successfully used to compare the chemosensory properties of mammalian and *Drosophila* TRPA1 isoforms in studies that have provided invaluable information about the structure-function relationship of these channels [33,37].

Initially, dTRPA1 was reported to be necessary in olfactory neurons of the basiconic sensilla of the antennae for the avoidance of citronellal odor through an indirect G-protein/PLC coupled mechanism, and that this compound has a very small direct stimulatory action on dTRPA1(B) [11]. Accordingly, citronellal was later reported to specifically activate dTRPA1(A) [14], and subsequent studies demonstrated activation of dTRPA1(C) and dTRPA1(D) as well. While our results support the activation of dTRPA1(A), (C) and (D), we also found activation of the dTRPA1(B) isoform. The concentrations of citronellal in natural settings are unclear, but the concentrations used in insect repellent formulations can be as high as 10–100% [50], which represent molar concentrations. These are 1000-fold or higher than the concentrations we used in our study (0.001–1 mM). Thus, our results may be relevant to understand the use of citronellal as insect repellent.

We based our comparison of the responses of dTRPA1-transfected cells to citronellal and menthol mainly on Ca^2+^-imaging data. This technique does not allow drawing conclusions about the efficacies of these compounds on the dTRPA1 isoforms, because it does not allow to correct for potential differences in the number of functional channels expressed in the plasma membrane. Therefore, the citronellal and menthol concentration-response data were also analyzed as values normalized to the amplitude of subsequent responses to AITC. The Ca^2+^-imaging experiments were designed to include a washout period between the highest concentration of citronellal or menthol and the application of AITC, which allowed intracellular Ca^2+^ to return to near baseline values. However, this design does not fully exclude the possibility of a partial channel desensitization/sensitization, which may lead to modulation of the responses to AITC. As such, the interpretation of the normalized data should consider the possibility of over- or under-estimations of the relative effects of citronellal and menthol. Furthermore, we previously showed that the amplitudes of Ca^2+^ responses to consecutive applications of weak and strong stimuli do not correlate across cells [51], and therefore the ratio of these amplitudes may not be an accurate measure of the relative strength of the stimuli. As concentrations above 1 mM gave rise to non-specific effects of citronellal and menthol, we faced the difficulty of not being able to fit the concentration-response data. Thus, our conclusions are based on sensitivity estimates defined as the minimal concentration at which the Ca^2+^ increase was detectable rather than EC_50_ values.

**Figure 7:**
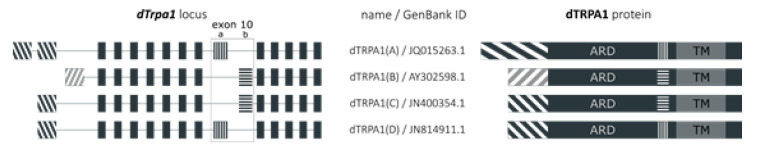
Schematic representation of dTRPA1 sequences and proteins. Diagram of the genomic locus of *dTrpa1* (**left**) resulting in different isoforms of dTRPA1 proteins (**right**). In the middle: the nomenclature that we maintain and the corresponding GenBank reference. ARD = ankyrin repeat domains, TM = trans-membrane segments.

Within these limitations, we found that the sensitivity of dTRPA1(B) to citronellal was lower compared to the other isoforms, but at the highest concentration tested the responses of cells transfected with this isoform were larger than for the other isoforms. dTRPA1(C) and dTRPA1(D) have identical *N*-termini and share most of this domain with dTRPA1(A). In contrast, dTRPA1(B) is markedly different in the distal *N*-terminal region compared to the other three isoforms (Figure 7 and Figure S3) [10]. This difference may account for the lower sensitivity of this isoform to citronellal.

In regard to menthol, the well-documented activation of mouse TRPA1 induced by this compound [31,33] can readily explain the implication of this channel in the aversive behavior of mice to menthol in a drinking assay [52]. In contrast, the scenario in *Drosophila* is not so clear, as the precise role of dTRPA1 in menthol-induced aversion during oviposition [40] and nocifensive responses of larvae towards direct contact [34] remained unexplored. In fact, the reported lack of stimulatory action of menthol on dTRPA1 [33], led to the suggestion that these channels are mediators of chemotransduction but not the actual menthol receptors [34]. On the other hand, our present results provide for a straightforward explanation for these apparently conflicting reports. Firstly, using an entirely different experimental setup, purely based in olfaction, we found that the olfactory-mediated avoidance of menthol by adult *Drosophila* flies depends largely on functional TRPA1, thus demonstrating the general role of this channel in the aversive responses to menthol. Secondly, we show that menthol activates all four *Drosophila* TRPA1 isoforms, although to very different extents. The sensitivity series we uncovered, (dTRPA1(A) > dTRPA1(C) = dTRPA1(D) ≫ dTRPA1(B)) is consistent with earlier studies reporting only on dTRPA1(B) [26,33]. Hence, we suggest that all isoforms may contribute to menthol-induced behaviors although in distinct concentration ranges and sensory cells.

Of note, we found that the avoidance to menthol was more susceptible to *dTrpA1* deletion than the response to citronellal, as for the latter significant responses were found in mutant flies. These results indicate that, at the concentration tested, dTRPA1 channels are critically required for avoidance of menthol and are consistent with the report of dual pathways necessary for the response to citronellal, i.e., a dTRPA1-dependent one and another dependent on olfaction [11].

Previous work showed that the sensitivity of mouse TRPA1 to menthol depends largely on serine and threonine residues located in the inner side of the TM5. Mutation of these residues drastically shifts the EC_50_ of menthol to about a ten-fold higher value in mouse TRPA1, and abrogates menthol responses in human TRPA1 [33]. However, all TM segments, including the TM5 segment, are identical among the dTRPA1 isoforms (Figure 7 and Figure S3). Therefore, other structure variations should account for the observed differences in sensitivity to menthol.

Another study showed that mutation of cysteine residues located between the ankyrin repeat domain (ARD) and the TM1 segment decreased (C641A) or increased (C621A and C665) the apparent sensitivity of human TRPA1 to menthol [53]. In dTRPA1, the region containing a corresponding residue, C694, (exon 10a) is spliced out in the dTRPA1(B) and dTRPA1(C) isoforms, while dTRPA1(A) and dTRPA1(D) retain it (Figure 7 and Figure S3) [4,21]. The comparison of the amino acid sequences of the dTRPA1 isoforms allows gaining insights into the structural determinants of the differences in sensitivity to menthol. First, dTRPA1(B) and dTRPA1(C), which both lack exon 10a and C694 but have different distal *N*-termini, display distinct sensitivities to menthol. Second, dTRPA1(C) and dTRPA1(D) differ only in exon 10 and have similar sensitivities to menthol. And third, the region surrounding C694 contains several cysteines, which are retained in all *Drosophila* isoforms, and are therefore unlikely to contribute to the differences in menthol sensitivity between the dTRPA1 isoforms [14,54]. Thus, as for citronellal, it seems that the main determinant of the significantly lower sensitivity of dTRPA1(B) is its distinctive distal *N*-terminus. It must be noticed, that none of the above-mentioned residues have been proven to contribute to an actual binding site for menthol in mammalian TRPA1 channels. Structurally, the cysteine-rich region between the TM1 and ARD is linked via the TRP-like domain to the TM4-TM5 linker (where the former-mentioned serine and threonine residues are located) [55]. These three segments may interact, shaping a tertiary structure critical for menthol sensitivity. Disturbing this tertiary structure by introducing mutations or by alternative splicing, could therefore alter menthol-induced activation. Alternatively, menthol may activate dTRPA1 channels by inducing mechanical perturbations in the plasma membrane, as it has been proposed for mammalian TRPA1 activation by this compound [33], trinitrophenol [56], primary alcohols [57], bacterial lipopolysaccharides (LPS) [17,19,58,59] and alkylphenols [60]. Notably, it was shown that HEK293T cells transfected with dTRPA1(B) were less responsive to LPS than cells transfected with dTRPA1(A). This further supports the notion that the dTRPA1(B) isoform is the least sensitive to non-electrophilic agonists.

We also found that application of HC-030031 has weaker inhibitory effects on dTRPA1 currents than previously reported for mammalian isoforms [61]. This is consistent with HC-030031 having species-dependent effects, inhibiting the human, mouse, chicken and green anole TRPA1 isoforms, but not those from the western clawed frog (fTRPA1) and zebrafish (zTRPA1) [61]. Moreover, our results indicate that HC-030031 has stronger inhibitory effects on the dTRPA1 currents elicited by citronellal than on those induced by menthol. For example, HC-030031 fully inhibited the citronellal effect on dTRPA1(A) current, but reduced the menthol-induced current only in about 50%. The latter observation is reminiscent of the previously reported weak action of this inhibitor on dTRPA1(A) currents induced by LPS [16]. This may indicate that the inhibitory action of HC-030031 on dTRPA1 channels is stimulus-dependent and that menthol and LPS activate the channels via similar mechanisms. In turn, this mechanism may be different from that operating for citronellal. Future studies are required to elucidate the mechanism of action of HC-030031 on dTRPA1 channels.

In conclusion, we here further illustrate that *dTrpA1* is implicated in the repellent action of citronellal and menthol on *D. melanogaster*, and demonstrate that all *Drosophila* dTRPA1 isoforms are activated by these compounds, albeit with large differences in sensitivity. The latter findings and the comparison of the distinct *N*-terminal structures of dTRPA1 isoforms may be instrumental for future mutation and chimeric studies aiming at unveiling the structure-function features of TRPA1 channels. In turn, this may serve to better understand the pathophysiological roles of these channels in flies and other animals, and may help in the design of strategies to use TRPA1 as target for pest control and for the pharmacological treatment of human diseases.

## 4. Materials and Methods

### 4.1. Drosophila Stocks

*Drosophila melanogaster* strains were raised on standard cornmeal/agar medium supplemented with dry yeast at 25 °C with a 12 h light/dark cycle. The wild type (WT) stock was a *w^1118^* strain. The *dTrpA1^1^* (BL26504) stock was obtained from the Bloomington Stock Center (Bloomington, IN, USA).

### 4.2. Direct Airborne Repellent Test (DART)

Around 40 flies (50% male, 50% female) were placed in a 14 cm diameter Petri dish covered with 1% agar. Dishes were rotated to prevent direction bias. Recordings started immediately after loading the vehicle and experimental compounds. To avoid the spread of the tested compounds (citronellal, menthol and respective vehicles) by the flies themselves, the compounds were applied on separate filter papers that were allowed to dry for 1 min before they were placed in an inverted lid of a 1.5 mL Eppendorf tube in the arena (see Figure 1 blue and green crosses in the arena). In this way the source of the olfactory stimulus was not accessible for the flies, and the olfactory stimulus originated from one fixed point in the arena. Thirty minutes after loading of the chemical compounds, the position of each fly was assessed in the arena. This was done by thresholding the image until each individual fly was selected. Subsequently each fly’s position as well as the position of the chemical compounds was exported as an X-Y coordinate. Then, the field for the vehicle and the experimental compound was set as the circular surface with the center at the X-Y position of the respective compound and a radius: $\frac{1}{2}\sqrt{{\left(x_{\mathrm{veh}}-x_{\exp}\right)}^{2}+{\left(y_{\mathrm{veh}}-y_{\exp}\right)}^{2}}$. The number of flies located in the field surrounding the experimental compound (Exp.) or vehicle (Veh.) were counted 30 min after applying the compounds, and the avoidance index (A.I.) was calculated as previously described [11] (Figure 1a).

### 4.3. Culture and Transfection of HEK293T Cells

Human embryonic kidney cells (HEK293T) were dispersedly seeded on 18 mm glass coverslips coated with poly-L-lysine (0.1 mg/mL) at a density of 20,000 cells/well and grown in Dulbecco’s modified Eagles medium containing 10% (*v*/*v*) fetal calf serum, 2 mM L-glutamine, 2 U/mL penicillin and 2 mg/mL streptomycin at 37 °C in a humidity-controlled incubator with 10% CO_2_. Cells were transiently transfected using Trans-IT-293 reagents (Mirus, Madison, MI, USA) with the different dTRPA1 isoforms cloned into the pCAGGS/IRES-GFP vector [62]. The A and B isoforms (GenBank accession numbers JQ015263 and AY302598, respectively) were kindly provided by Dr. Paul Garrity (Brandeis University, USA), and the C and D isoforms (GenBank accession numbers JN400354 and JN814911, respectively) were isolated from total RNA extracted from *D. melanogaster* larvae, kindly donated by Dr. Alessia Soldano (VIB, Belgium), as described elsewhere [21].

### 4.4. Intracellular Ca^2+^ Imaging

For intracellular Ca^2+^ imaging measurements cells were incubated at 37 °C with 2 µM Fura2-AM ester for 30 min before the recordings. The extracellular solution contained (in mM): 140 NaCl, 5 KCl, 10 HEPES, 2 CaCl_2_, 2 MgCl_2_, 10 glucose; pH titrated to 7.4 with NaOH. Fluorescent signals were evoked during alternating illumination at 340 and 380 nm using a Lambda XL illuminator (Sutter Instruments, Novato, CA, USA), and recorded using an Orca Flash 4.0 camera (Hamamatsu Photonics Belgium, Mont-Saint-Guibert, Belgium) on a Nikon Eclipse Ti fluorescence microscope (Nikon Benelux, Brussels, Belgium). The imaging data was recorded and analyzed using NIS-elements software (Nikon) at 23 °C. Fluorescence was measured during excitation at 340 and 380 nm, and after correction for the individual background fluorescence signals, the ratio of the fluorescence at both excitation wavelengths (F340/F380) was monitored. Intracellular Ca^2+^ concentrations were calculated as previously described [63]. In all experiments transfected cells were identified by GFP expression and the presence of responses to the application of the TRPA1 agonist allyl isothiocyanate (100 µM; Sigma-Aldrich, Bornem, Belgium) at the end of the experiment.

### 4.5. Patch-Clamp Experiments

Whole-cell currents were measured at 23 °C with an EPC-10 patch-clamp amplifier and the software Patchmaster (HEKA electronic, Lambrecht, Germany). Currents were digitally filtered at 3 kHz, acquired at 20 kHz and stored for off-line analysis on a personal computer. The cell membrane capacitance (C_m_ = 12.2 ± 0.3 pF; n = 119), and the series resistance (always below 4 MΩ) were determined using the built-in compensation circuits of the EPC-10 amplifier. R_s_ was electronically compensated up to 50% without ringing and was continually monitored during the experiment. The liquid junction potential was compensated before establishing the gigaseal using the built-in compensation circuits of the EPC-10 amplifier. Currents were recorded in an extracellular solution containing (in mM): 140 NaCl, 5 KCl, 10 HEPES, 2 CaCl_2_, 2 MgCl_2_, 10 glucose; pH titrated to 7.4 with NaOH. The extracellular solutions were applied by gravity as previously described [64]. The pipette solution contained (in mM): 120 Cs-Aspartate, 5 EGTA, 10 HEPES, 1 MgCl_2_; pH titrated to 7.2 with CsOH. Non-transfected HEK293T cells were used as control. Currents were elicited using a 200 ms voltage ramp from −125 mV to +125 mV every 2 s from a holding potential of 0 mV. The rectification index of the currents was calculated as −I(+75 mV)/I(−75 mV). NMDG^+^ (*N*-methyl-d-glucamine-Cl) was used to monitor the size of the leak currents during the recordings as previously described [44].

### 4.6. Data Analysis

Electrophysiological data were analyzed using the softwares WinASCD (Guy Droogmans, KU Leuven) and Origin (OriginLab Corporation, Northamptom, MA, USA). Origin was also used for calculations, statistical analysis and data display. For the fly behavior experiments, statistical significance of difference of each condition compared to the value 0 (= absence of avoidance towards the experimental compound) was calculated using the one-sample *t*-test vs. 0. The statistical significance between two distributions was measured using the unpaired Student’s *t*-test. For the basal currents and rectification index, statistical significance of difference between isoforms and the non-transfected cells (= negative control), was calculated using the Kruskal-Wallis test. For the rectification index, statistical significance of difference compared to the value 1 (= absence of current rectification), was calculated using the Wilcoxon signed rank test. We used the One Sample Wilcoxon Signed Rank Test to determine, for each dTRPA1 isoform, the concentrations of citronellal and menthol for which the change in intracellular Ca^2+^ concentration was significantly larger than zero. We used the Kruskal-Wallis ANOVA to determine whether the responses to 1 mM citronellal or 1 mM menthol were different across dTRPA1 isoforms. For the whole cell currents measured in the presence of agonists, we calculated the current density by dividing whole-cell currents by the cell’s capacitance. Then, we performed baseline subtraction and the statistical significance of difference of the ΔI (pA/pF) compared to 0 (= absence of current change) was calculated using the one-sample *t*-test vs. 0. The significance of difference between currents elicited by either agonist (citronellal or menthol) in the absence and presence of the TRPA1-blocker HC030031, was measured using the one tailed paired sample *t*-test. All data are expressed as means ± SEM. A value of *p* ≤ 0.05 was considered as statistically significant.

## Data Availability

Data is contained within the article or Supplementary Materials.

## Supplementary Materials

The following are available online at https://www.mdpi.com/article/10.3390/ijms222010997/s1, Figure S1: Original fly behavioral data., Figure S2: Analysis of fly behavioral data. Figure S3: *Drosophila melanogaster* TRPA1 isoforms, Video S1: WT_citronellal, Video S2: TrpA1KO_citronellal, Video S1: WT_menthol, Video S1: TrpA1KO_menthol.

## Abbreviations

A.I.	avoidance index
AITC	Allyl isothiocyanate
ARD	ankyrin repeat domain
DART	Direct airborne repellent test
DMSO	dimethyl sulfoxide
HEK293T	human embryonic kidney cells 293T
NMDG^+^	N-methyl-D glucamine
PLC	phopholipase C
TM	transmembrane segment
TRPA1	transient receptor potential ankyrin 1
WT	wild type
