# Supplementary material for: Activation of Drosophila melanogaster TRPA1 Isoforms by Citronellal and Menthol
Source: Int J Mol Sci. 2021 Oct 12;22(20):10997. doi: 10.3390/ijms222010997 (PMC8541009; doi:10.3390/ijms222010997)
Supplement: Supplementary file 1 [file ijms-22-10997-s001.zip › ijms-1385387-supplementary.pdf]

Supplementary Information

Activation of *Drosophila melanogaster* TRPA1 isoforms by citronellal and menthol

Brett Boonen, Justyna Startek, Alina Milici, Alejandro López-Requena, Melissa Beelen, Patrick Callaerts and Karel Talavera

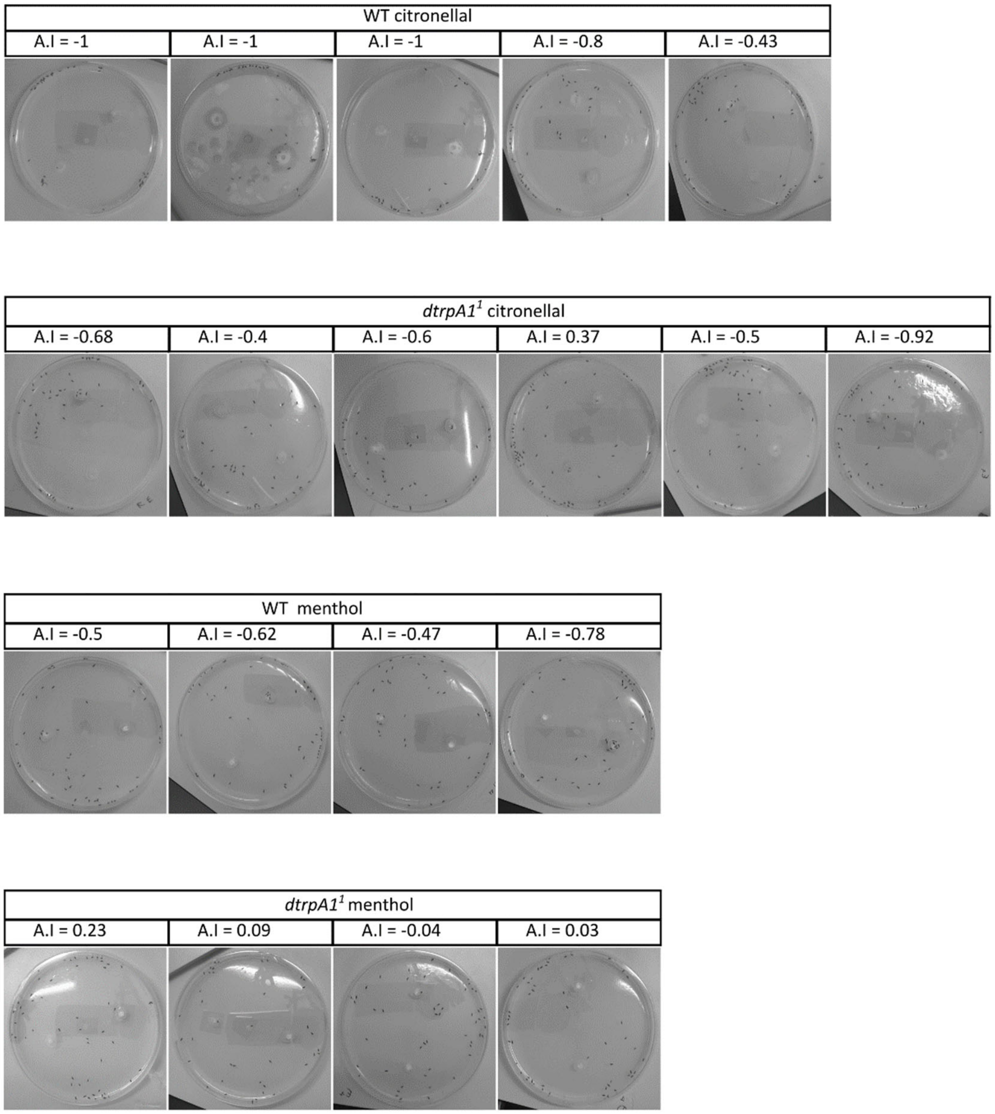

Figure S1. Original fly behavioral data. Photographs of the direct airborne repellent test (DART) performed with adult WT and *dTrpA1*^{1} flies exposed for 30 min to vehicle and citronellal or menthol. The avoidance index towards the corresponding test compounds (A.I.) are given above each image.

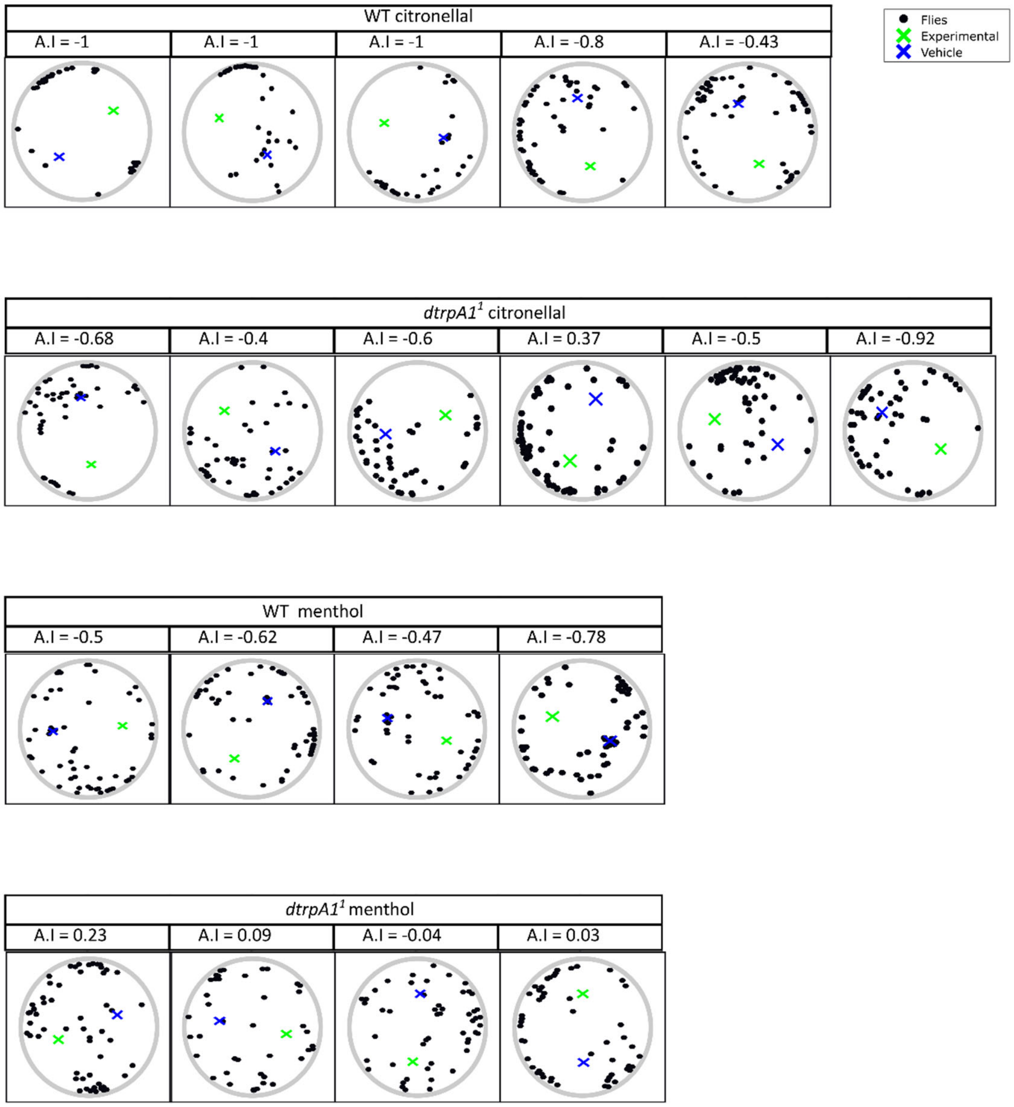

Figure S2. Analysis of fly behavioral data. Images obtained from the analysis of the photographs of the direct airborne repellent test (DART), corresponding one-to-one with the pictures shown in Figure S1. The positions of the vehicle and test compounds (citronellal or menthol) are indicated by the blue and green crosses, respectively. The black dots indicate the positions of the flies detected by imaging analysis. The avoidance index towards the corresponding test compounds (A.I.) are given above each image.

Supplemental Videos S1, S2, S3 and S4 available at: https://zenodo.org/record/5563012#.YWSoWxpBzb0

Figure S3. *Drosophila melanogaster* TRPA1 isoforms

A: 1251 aa (Nature. 2011 Dec 4;481(7379):76-80)
D: 1232 aa (Cell Rep. 2012 Jan 26;1(1):43-55)
C: 1231 aa (Cell Rep. 2012 Jan 26;1(1):43-55)
B: 1197 aa (Nature. 2003 Jun 19;423(6942):822-3)

Residues isoform-specific
Mutation
Transmembrane
Intramembrane (pore-forming)

```
A       MITAPATATERNQSSKSTRMPKLYNGVYSGQCGALSPPDLMEAQPKLLPKPRSNSSGSTG
D       -------------------MPKLYNGVYSGQCGALSPPDLMEAQPKLLPKPRSNSSGSTG
C       -------------------MPKLYNGVYSGQCGALSPPDLMEAQPKLLPKPRSNSSGSTG
B       ------------------------------------------------------MTSGDK

A       RNSKYWIFSMIIERSAGPKRIEIDGDDADTPLEAILPAEPPAEVCLLRDSPFRILR
D       RNSKYWIFSMIIERSAGPKRIEIDGDDADTPLEAILPAEPPAEVCLLRDSPFRILR
C       RNSKYWIFSMIIERSAGPKRIEIDGDDADTPLEAILPAEPPAEVCLLRDSPFRILR
B       ETPKREDFASALRFLMGGCAREPEMTAMAPLNLPKKWARILRMSSTPKIPIVDYLE

A       AAESGNLDDFKRLFMADNSRIALKDAKGRTAAHQAAARNRVNILRYIRDQNGDFNAK
D       AAESGNLDDFKRLFMADNSRIALKDAKGRTAAHQAAARNRVNILRYIRDQNGDFNAK
C       AAESGNLDDFKRLFMADNSRIALKDAKGRTAAHQAAARNRVNILRYIRDQNGDFNAK
B       AAESGNLDDFKRLFMADNSRIALKDAKGRTAAHQAAARNRVNILRYIRDQNGDFNAK
        *********************************************************

A       DNAGNTPLHIAVESDAYDALDYLLSIPVDTGVLNEKKQAPVHLATELNKVKSLRVMGQYR
D       DNAGNTPLHIAVESDAYDALDYLLSIPVDTGVLNEKKQAPVHLATELNKVKSLRVMGQYR
C       DNAGNTPLHIAVESDAYDALDYLLSIPVDTGVLNEKKQAPVHLATELNKVKSLRVMGQYR
B       DNAGNTPLHIAVESDAYDALDYLLSIPVDTGVLNEKKQAPVHLATELNKVKSLRVMGQYR
        ************************************************************

A       NVIDIQQGGEHGRTALHLAAIYDHEECARILITEFDACPRKPCNNGYYPIHEAAKNASSK
D       NVIDIQQGGEHGRTALHLAAIYDHEECARILITEFDACPRKPCNNGYYPIHEAAKNASSK
C       NVIDIQQGGEHGRTALHLAAIYDHEECARILITEFDACPRKPCNNGYYPIHEAAKNASSK
B       NVIDIQQGGEHGRTALHLAAIYDHEECARILITEFDACPRKPCNNGYYPIHEAAKNASSK
        ************************************************************

A       TMEVFFQWGEQRGCTREEMISFYDSEGNVPLHSAVHGGDIKAVELCLKSGAKISTQQHDL
D       TMEVFFQWGEQRGCTREEMISFYDSEGNVPLHSAVHGGDIKAVELCLKSGAKISTQQHDL
C       TMEVFFQWGEQRGCTREEMISFYDSEGNVPLHSAVHGGDIKAVELCLKSGAKISTQQHDL
B       TMEVFFQWGEQRGCTREEMISFYDSEGNVPLHSAVHGGDIKAVELCLKSGAKISTQQHDL
        ************************************************************

A       STPVHLACAQGAIDIVKLMFEMQPMEKRLCLSCTDVQKMTPLHCASMFDHPDIVSYLVAE
D       STPVHLACAQGAIDIVKLMFEMQPMEKRLCLSCTDVQKMTPLHCASMFDHPDIVSYLVAE
C       STPVHLACAQGAIDIVKLMFEMQPMEKRLCLSCTDVQKMTPLHCASMFDHPDIVSYLVAE
B       STPVHLACAQGAIDIVKLMFEMQPMEKRLCLSCTDVQKMTPLHCASMFDHPDIVSYLVAE
        ************************************************************

A       GADINALDKEHRSPLLLAASRSGWKTVHLLIRLGACISVKDAAARNVLHFVIMNGGRLTD
D       GADINALDKEHRSPLLLAASRSGWKTVHLLIRLGACISVKDAAARNVLHFVIMNGGRLTD
C       GADINALDKEHRSPLLLAASRSGWKTVHLLIRLGACISVKDAAARNVLHFVIMNGGRLTD
B       GADINALDKEHRSPLLLAASRSGWKTVHLLIRLGACISVKDAAARNVLHFVIMNGGRLTD
        ************************************************************

A       FAEQVANCQTQAQLKLLLNEKDSMGCSPLHYASRDGHIRSLENLIRLGACINLKNNNNES
D       FAEQVANCQTQAQLKLLLNEKDSMGCSPLHYASRDGHIRSLENLIRLGACINLKNNNNES
C       FAEQVANCQTQAQLKLLLNEKDSMGCSPLHYASRDGHIRSLENLIRLGACINLKNNNNES
B       FAEQVANCQTQAQLKLLLNEKDSMGCSPLHYASRDGHIRSLENLIRLGACINLKNNNNES
        ************************************************************

A       PLHFAARYGRYNTVRQLLDSEKGSFIINESDGAGMTPLHISSQQGHTRVVQLLLNRGALL
D       PLHFAARYGRYNTVRQLLDSEKGSFIINESDGAGMTPLHISSQQGHTRVVQLLLNRGALL
C       PLHFAARYGRYNTVRQLLDSEKGSFIINESDGAGMTPLHISSQQGHTRVVQLLLNRGALL
B       PLHFAARYGRYNTVRQLLDSEKGSFIINESDGAGMTPLHISSQQGHTRVVQLLLNRGALL
        ************************************************************
```

```
A       HRDHTGRNPLQLAAMSGYTETIELLHSVHSHLLDQVDKDGNTALHLATMENKPHAISVLM
D       HRDHTGRNPLQLAAMSGYTETIELLHSVHSHLLDQVDKDGNTALHLATMENKPHAISVLM
C       HRDHTGRNPLQLAAMSGYTETIELLHSVHSHLLDQVDKDGNTALHLATMENKPHAISVLM
B       HRDHTGRNPLQLAAMSGYTETIELLHSVHSHLLDQVDKDGNTALHLATMENKPHAISVLM
        ************************************************************

A       SMGCKLVYNVLDMSAIDYAIYYKYQEAALAMVTHEERANEVMALRSDKHPCVTLALIASM
D       SMGCKLVYNVLDMSAIDYAIYYKYPEAALAMVTHEERANEVMALRSDKHPCVTLALIASM
C       SMGCKLVYNVLDMSAIDYAIYYKYPEAALAMVTHEERANEVMALRSDKHPCVTLALIASM
B       SMGCKLVYNVLDMSAIDYAIYYKYPEAALAMVTHEERANEVMALRSDKHPCVTLALIASM
        ************************ ***********************************

A       PKVFEAVQDKCITKANCKKDSKSFYIKYSFAFLQCPFMFAKIDEKTGESITTASPIPLPAL
D       PKVFEAVQDKCITKANCKKDSKSFYIKYSFAFLQCPFMFAKIDEKTGESITTASPIPLPAL
C       PKVFEAVQDKCITKANCKKDSKSFYIKYSFWPYQKTPEQIEAKRKEFNDPKWRPAPLAVV
B       PKVFEAVQDKCITKANCKKDSKSFYIKYSFAFLQCPFMFAKIDEKTGESITTASPIPLPAL
        ******************************

A       NTMVTHGRVELLAHPLSQKYLQMKWNSYGKYFHLANLLIYSIFLVFVTIYSSLMMNNI
D       NTMVTHGRVELLAHPLSQKYLQMKWNSYGKYFHLANLLIYSIFLVFVTIYSSLMMNNI
C       NTMVTHGRVELLAHPLSQKYLQMKWNSYGKYFHLANLLIYSIFLVFVTIYSSLMMNNI
B       NTMVTHGRVELLAHPLSQKYLQMKWNSYGKYFHLANLLIYSIFLVFVTIYSSLMMNNI
        **********************************************************

A       ELKAGDNKTMSQYCNMGWEQLTMNLSQNPSVASQIRLDSCEERINRTTAILFCAVVIVVY
D       ELKAGDNKTMSQYCNMGWEQLTMNLSQNPSVASQIRLDSCEERINRTTAILFCAVVIVVY
C       ELKAGDNKTMSQYCNMGWEQLTMNLSQNPSVASQIRLDSCEERINRTTAILFCAVVIVVY
B       ELKAGDNKTMSQYCNMGWEQLTMNLSQNPSVASQIRLDSCEERINRTTAILFCAVVIVVY
        ************************************************************

A       ILLNSMRELIQIYQQKLHYILETVNLISWVLYISALVMVTPAFQPDGGINTIHYSAASIA
D       ILLNSMRELIQIYQQKLHYILETVNLISWVLYISALVMVTPAFQPDGGINTIHYSAASIA
C       ILLNSMRELIQIYQQKLHYILETVNLISWVLYISALVMVTPAFQPDGGINTIHYSAASIA
B       ILLNSMRELIQIYQQKLHYILETVNLISWVLYISALVMVTPAFQPDGGINTIHYSAASIA
        ************************************************************

A       VFLSWFRLLLFLQRFDQVGIYVVMFLEILQTLIKVLMVFSILIIAFGLAFYILLSKIIDP
D       VFLSWFRLLLFLQRFDQVGIYVVMFLEILQTLIKVLMVFSILIIAFGLAFYILLSKIIDP
C       VFLSWFRLLLFLQRFDQVGIYVVMFLEILQTLIKVLMVFSILIIAFGLAFYILLSKIIDP
B       VFLSWFRLLLFLQRFDQVGIYVVMFLEILQTLIKVLMVFSILIIAFGLAFYILLSKIIDP
        ************************************************************

A       QPNHLSFSNIPMSLLRTFSMMLGELDFVGTYVNTYYRDQLKVPMTSFLILSVFMILMPIL
D       QPNHLSFSNIPMSLLRTFSMMLGELDFVGTYVNTYYRDQLKVPMTSFLILSVFMILMPIL
C       QPNHLSFSNIPMSLLRTFSMMLGELDFVGTYVNTYYRDQLKVPMTSFLILSVFMILMPIL
B       QPNHLSFSNIPMSLLRTFSMMLGELDFVGTYVNTYYRDQLKVPMTSFLILSVFMILMPIL
        ************************************************************

A       LMNLLIGLAVGDIESVRRNAQLKRLAMQVVLHTELERKLPHVWLQRVDKMELIEYPNETK
D       LMNLLIGLAVGDIESVRRNAQLKRLAMQVVLHTELERKLPHVWLQRVDKMELIEYPNETK
C       LMNLLIGLAVGDIESVRRNAQLKRLAMQVVLHTELERKLPHVWLQRVDKMELIEYPNETK
B       LMNLLIGLAVGDIESVRRNAQLKRLAMQVVLHTELERKLPHVWLQRVDKMELIEYPNETK
        ************************************************************

A       CKLGFCDFILRKWFSNPFTEDSSMDVISFDNNDDYINAELERQRRKLRDISRMLEQQHHL
D       CKLGFCDFILRKWFSNPFTEDSSMDVISFDNNDDYINAELERQRRKLRDISRMLEQQHHL
C       CKLGFCDFILRKWFSNPFTEDSSMDVISFDNNDDYINAELERQRRKLRDISRMLEQQHHL
B       CKLGFCDFILRKWFSNPFTEDSSMDVISFDNNDDYINAELERQRRKLRDISRMLEQQHHL
        ************************************************************

A       VRLIVQKMEIKTEADDVDEGISPNELRSVVGLRSAGGNRWNSPRVRNKLRAALSFNKSM
D       VRLIVQKMEIKTEADDVDEGISPNELRSVVGLRSAGGNRWNSPRVRNKLRAALSFNKSM
C       VRLIVQKMEIKTEADDVDEGISPNELRSVVGLRSAGGNRWNSPRVRNKLRAALSFNKSM
B       VRLIVQKMEIKTEADDVDEGISPNELRSVVGLRSAGGNRWNSPRVRNKLRAALSFNKSM
        ***********************************************************
```
